# Treatment outcome of pediatric tuberculosis in eastern Ethiopia

**DOI:** 10.3389/fped.2022.966237

**Published:** 2022-08-11

**Authors:** Fitsum Weldegebreal, Zelalem Teklemariam, Habtamu Mitiku, Tamrat Tesfaye, Aklilu Abrham Roba, Fikru Tebeje, Abiyot Asfaw, Mahantash Naganuri, Bahubali Jinnappa Geddugol, Frehiwot Mesfin, Ibsa Mussa Abdulahi, Hilina Befikadu, Eden Tesfaye

**Affiliations:** ^1^College of Health and Medical Science, Haramaya University, Harar, Ethiopia; ^2^College of Social Sciences and Humanities, Haramaya University, Dire Dawa, Ethiopia; ^3^College of Natural and Computational Sciences, Haramaya University, Dire Dawa, Ethiopia

**Keywords:** treatment outcome, pediatric tuberculosis, eastern Ethiopia, retrospective study, a nine years

## Abstract

**Background:**

Children are more vulnerable to developing active *Mycobacterium tuberculosis* infection which causes significant morbidity and mortality. However, the contribution of childhood tuberculosis and its treatment outcomes have not been well documented, and no research has been conducted in eastern Ethiopia.

**Objective:**

This study aimed to assess the treatment outcome and its predictors of pediatric tuberculosis in eastern Ethiopia from September 1, 2017 to January 30, 2018.

**Methods:**

A retrospective study was conducted in eight selected hospitals in eastern Ethiopia. Data on 2002 children with tuberculosis was extracted by using the standard checklist of the national tuberculosis treatment format. Treatment outcomes were determined according to the standard definitions of the National Tuberculosis and Leprosy Control Programme. Data were entered into Epi Data software version 3.1 and exported to Statistical Package for Social Science (SPSS) version 20 for analysis. Bivariable and multivariable regression analyses were carried out to examine the associations between dependent and independent variables. A P-value of <0.05 was considered statistically significant.

**Result:**

The overall successful treatment rate was 1,774 (88.6%) [95% confidence interval (CI): (80.59–97.40)]. A total of 125 (6.2%), 1,648 (82.3%), 59 (2.9%), and 19 (0.9%) children with tuberculosis (TB) were cured, completed, defaulted, and died, respectively. A high number of defaulters and deaths were reported in the age group <10 years. More children with smear-positive pulmonary TB (74.4%) were cured, while smear-negative tuberculosis had higher treatment completion rates. Being male in sex (adjusted odds ratio (AOR): 0.71, 95% CI: 0.53, 0.96) and those with human immunodeficiency virus **(**HIV) positive sero status (AOR: 0.51, 95% CI: 0.29, 0.90) had a lower chance of a successful treatment outcome.

**Conclusion:**

In this study, thee treatment success rate was higher than the recent World Health Organization report. Those males and HIV seropositive status were less likely to have a successful treatment outcome. Therefore, efforts should be made by each health institution in eastern Ethiopia by giving emphasis on male and HIV-positive individuals.

## Introduction

Tuberculosis (TB) remained the leading cause of death due to a single infectious pathogen. In 2019, an estimated 10.0 million people developed TB, with 1.2 million TB deaths among HIV-negative people and 208, 000 deaths among HIV-positive people (1). The World Health Organization (WHO) regions with the highest number of TB patients were found Africa (25%), South-East Asia (44%), and the Western Pacific (18%) children under the age of 15 are another important subset of missing people with tuberculosis. Children have greater diagnostic and treatment gaps than adults (1). Children are vulnerable to *Mycobacterium tuberculosis* infection in the community and are more likely to develop the active disease if infected at a young age (2). Approximately 1 million cases of TB in children are predicted to occur globally. Over 75% of children with tuberculosis are from the 22 high-burden countries (3, 4) whereas a small proportion (~5%) is from low-burden countries (5). It was estimated to cause 74,000 to 130,000 deaths per year, making it one of the top ten causes of death in children (4, 5).

Lack of evaluation, poor treatment linkage, death, and a high loss to follow-up are the primary causes of poor treatment outcomes. Unidentified or additional drug resistance is one of the underlying causes of poor treatment outcomes (6). In addition, insufficient documentation and reporting, poor drug formulation alternatives, caregiver availability/capacity for treatment, and recurring stock-outs of the few pharmacological options are all challenges for children (1). By 2019, only 30% of the 3.5 million 5-year targets for tuberculosis treatment in children had been achieved, and only 8% of the 115,000 targets for children with rifampicin resistance/multidrug resistance-TB.- Gaps in data collection and adequate and consistent disaggregation continue to have a negative impact on identification, treatment, programming, and resource allocation for children under the age of 15 (1). In Ethiopia, children with TB account for 16.1% of the national TB burden (4).

Childhood TB is a marker of recent transmission in a population; additionally, children are the primary victims of a poor tuberculosis control program (7). However, the highest priority has been given mostly to adult infectious TB cases. However, the management and prevention of TB in children are relatively neglected (4, 7).

Childhood tuberculosis surveillance data is critical for defining the disease's epidemiology and identifying predictors of poor treatment results. The World Health Organization recommends that children with tuberculosis be treated and notified through the national tuberculosis control program (3). However, in Ethiopia, as in other resource-constrained countries, such reports are primarily limited to adults infected with tuberculosis. Furthermore, except a few studies (8, 9), the contribution of childhood tuberculosis and its treatment outcomes is not well documented and no research has been conducted in eastern Ethiopia. Therefore, this study tried to assess the treatment outcome and its predictors for childhood tuberculosis in eastern Ethiopia.

## Methods and materials

### Study area and period

A retrospective cross-sectional study was conducted in eight selected eastern Ethiopian hospitals from September 1, 2017 to January 30, 2018. The selected hospitals were Jugal and Hiwot Fana Specialized University Hospitals from Harar, Karamara hospital from Jigjiga, Dilchora and Sabian hospitals from Dire Dawa, Dader hospital from Dader, and Chiro hospitals from West Hararghe. Harar is located in the eastern part of Ethiopia, 526 kilometers away from the capital city, Addis Ababa. The town has a projected total population of 203,438 in 2010. The town has four governmental hospitals, two private hospitals, and four health centers (10). Dire Dawa is the administrative city, which is located 515 kilometers from Addis Ababa on the Addis Ababa–Djibouti railroad in the eastern part of Ethiopia. The city has a population of 44,000 according to the 2015 projected report of the Ethiopian Central Statistical Agency (CSA). Jigjiga town is the capital city of the Ethiopian Somali Regional state which is located 625 kilometers from Addis Ababa and 100 kilometers from Harar City. The town has a population of 159,300 according to the 2015 projected report of the Ethiopian Central Statistical Agency (CSA) (11). Dader is one of the woredas in the Oromia Region of Ethiopia. According to the 2007 national census, this woreda had a total population of 242,140 people. Dader has one hospital which provides services to the surrounding community, including TB/MDR-TB services. Chiro/ Asebe Teferi is the administrative city of the West Hararghe Zone with a 49,500 population according to the 2015 projected report (11).

### Sample size determination

Two thousand two child TB patients registered for treatment from 2008/09 to 2016/17 at each health institute and who had complete TB treatment outcome records were retrieved.

### Data collection methods

Six trained nurses extracted information from each health facility's TB unit registers using a standard extraction checklist and the national TB treatment format, and the data collection process was supervised by two supervisors. Age and sex-specific demographics, TB types, smear results (baseline and follow-up for smear-positive PTB patients), TB categories, HIV sero status, and treatment outcomes were extracted using the checklist. Treatment outcomes of success (cured and completed treatment) and poor or unsatisfactory outcomes (failure, transfer out, default, and death) were measured after completing the standard anti-TB regimen.

### Method of data analysis

After checking for completeness and consistency of the collected information, the data were double entered into Epi data software version 3.1 and exported to SPSS version 20 for analysis. The socio-demographic and clinical characteristics of study participants were described using descriptive statistical analysis. Bivariable and multivariable logistic regression analyses were used to determine the relationship between dependent and independent variables. All explanatory variables with a *P*-value ≤ of 0.25 in the bi-variable analysis was included in a multivariable logistic regression model. A model of fitness was checked by the Hosmer and Lemeshow test. A *P*-value of <0.05 was considered as statistically significant.

### Data quality control

Data collectors were trained and the raw data was checked every day for completeness and consistency during and after data collection (before entry into a database). An adequate sample size was included from each study site. In addition, data were collected following strict inclusion and exclusion criteria. Moreover, to avoid data entry errors, the collected data were double entered into Epi Data software version 3.1.

## Results

### Socio-demographic characteristics

A total of 2,002 children who had completed information and registered for TB treatment in selected health facilities in eastern Ethiopia from 2008/09 to 2016/17 were included. The mean age of the study participants was 8.07 (SD+/- 4.6) and the range was 1–17 years. About 61.3%, 34.8%, 97.3%, 5.1%, and 61.1% were male in gender, age group (5–9 years), urban dwellers, HIV positive, and smear-negative pulmonary TB (SNPTB), respectively (Table 1).

**Table 1:** Sociodemographic characteristics of children TB patients attending treatment in selected health institutions of eastern Ethiopia, 2017 (*n* = 2,002).

**Variable**			**Number (%)**
Sex	Male		1,228 (61.3)
	Female		774 (38.7)
Age (in years)	0–4		515 (25.7)
	5–9		696 (34.8)
	10–14		557 (27.8)
	15–17		234 (11.7)
Resident	Urban		1,947 (97.3)
	Rural		55 (2.7)
HIV sero status of patients	Positive		103 (5.1)
	Negative		918 (45.9)
	Unknown		982 (49.1)
Type of TB	PTB	SPPTB	167 (8.3)	**1,391/2,002** **=69.4**
		SNPTB	1,224 (61.1)	
	EPTB		611 (30.5)
Type of TB patient	New		1,996 (99.7)
	Retreatment**		6 (0.3)

^**^Reasons for re-treatment was relapse.

Among extrapulmonary TB (EPTB); 52.1% (318/611), 25.5% (156/1611) and 22.4% (137/611) were lymph node, pleural and uncategorised types of TB, respectively. Males, urban dwellers, and HIV seronegative children had a higher prevalence of all TB forms; with smear-positive pulmonary TB (SPPTB) in the age group of 10–14, SNPTB and EPTB in the age group of 5–9. There was a high number of SNPTB (36.8%) in the age group of 5–9 and SPPTB in those with unknown HIV status (55.1%) (*p* < 0.05) (Table 2).

**Table 2:** Tuberculosis types distribution by study participant's characteristics attending treatment in selected health institutions of eastern Ethiopia, 2017.

**Variable**		**SPPTB No (%)**	**SNPTB No (%)**	**EPTB No (%)**	***P*-values**
Sex	Male	103 (61.7)	749 (61.2)	376 (61.5)	**0.985**
	Female	64 (38.3)	475 (38.8)	235 (38.5)	
Age	0–4	35 (21.0)	273 (22.3)	207 (33.9)	**0.001**
	5–9	45 (26.9)	451 (36.8)	200 (32.7)	
	10–14	49 (29.3)	369 (30.1)	139 (22.7)	
	15–17	38 (22.8)	131 (10.7)	65 (10.6)	
Resident	Urban	164 (98.2)	1,183 (96.7)	600 (98.2)	**0.118**
	Rural	3 (1.8)	41 (3.3)	11 (1.8)	
HIV sero status of patients	Positive	-	76 (6.2)	26 (4.3)	**0.007**
	Negative	75 (44.9)	556 (45.4)	287 (47.0)	
	Unknown	92 (55.1)	592 (48.4)	298 (48.8)	

Out of 167 children who were initially smear positive, 137 had their sputum examined after two months, and 2.9%(4/137) were positive. At the end of the 5th month, 2.2% (3/137) were positive and at the end of the 6th months, three cases were positive, with one case referred to the Multi-Drug Resistance treatment center.

### Magnitude of treatment outcome

The overall successful treatment rate among children infected with TB was 1774/2002 (88.6%) (95% CI: 80.59–97.40). Females 700/774 (90.4%), the age group of 15–17 211/234 (90.2%), rural dwellers 51/55 (92.7%), SPPTB 153/167 (91.6%), new TB category 1768/1996 (88.6%), and unknown HIV sero status 880/982(89.6%) had the highest treatment success. A total of 125/2002 (6.2%) and 1,648/2002 (82.3%) children with TB were cured and completed their treatment, respectively. A total of 59/2002 (2.9%) and 19/2002 (0.9%) of children with TB defaulted and died, respectively. And a total of three children with TB failed their treatment.

A higher number (83.5%) of female children completed their treatment. According to age, the highest percentage of children cured were found in the age group of 15–17, while those who completed treatment were found in the age group of 5–9. A high number of defaulters were found in the age group 10–14 years while deaths in 0–4 and 15–17 years of age. About 6.3% of children from urban areas were cured, whereas larger treatment completions were found in those from rural areas (87.3%). Besides, higher defaulters, died and failure cases were reported from urban dwellers. On the other hand, more children with SPPTB (74.9%) were cured (Table 3). From EPTB patients, high unsuccessful treatment was reported among children with lymph node and pleural TB, but the magnitude was not statistically significant (*p* > 0.05) (Table 4).

**Table 3:** Treatment outcome by socio-demographic characteristics and types of tuberculosis among children TB patients attending treatment in selected health institutions of eastern Ethiopia, 2017 (*n* = 2,002).

**Variable**			**TB treatment outcome**						
			**Successful**		**Poor/un successful**				
			**Cured *N* (%)**	**Completed *N* (%)**	**Defaulted *N* (%)**	**Died *N* (%)**	**Transfer out *N* (%)**	**Transfer to MDR unit *N* (%)**	**Failure *N* (%)**
Sex	Male		71 (5.8)	1,002 (81.6)	44 (3.6)	9 (0.7)	99 (8.1)	-	3 (0.2)
	Female		54 (7.0)	646 (83.5)	15 (1.9)	10 (1.3)	48 (6.2)	1 (0.1)	-
Age	0–4		23 (4.5)	427 (82.9)	22 (4.3)	9 (1.7)	34 (6.6)	-	-
	5–9		35 (5.0)	581 (83.5)	17 (2.4)	3 (0.4)	59 (8.5)	-	1 (0.1)
	10–14		43 (7.7)	453 (81.3)	18 (3.2)	3 (0.5)	39 (7)	1 (0.2)	-
	15–17		24 (10.3)	187 (79.9)	2 (0.9)	4 (1.7)	15 (6.4)	-	2 (0.9)
Residence	Urban		122 (6.3)	1,600 (82.2)	59 (3)	19 (1)	143 (7.3)	1 (0.1)	3 (0.2)
	Rural		3 (5.5)	48 (87.3)	-	-	4 (7.3)	-	-
Type of TB	PTB	SPPTB	125 (74.9)	28 (16.8)	10 (6)	-	-	1 (0.6)	3 (1.8)
		SNPTB	NA	1,091 (89.1)	28 (2.3)	10 (0.8)	95 (7.8)	-	-
	EPTB		NA	529 (89.6)	21 (3.4)	9 (1.5)	52 (8.5)	-	-
Category of TB patient	New		125 (6.3)	1,643 (82.3)	59 (3)	19 (1)	147 (7.4)	-	3 (0.1)
	Retreatment		-	5 (83.3)	-	-	-	1 (16.7)	-
HIV sero status of patients	Positive		-	84 (82.4)	-	-	18 (17.6)	-	-
	Negative		56 (6.1)	753 (82.0)	27 (2.9)	8 (0.9)	73 (8.0)	1 (0.1)	-
	Unknown		69 (7.0)	811 (82.6)	32 (3.3)	11 (1.1)	56 (5.7)	-	3 (0.3)

NA, not applicable.

**Table 4:** Treatment outcome of pediatrics EPTB among children attending treatment in selected health institutions of eastern Ethiopia, 2017 (*n* = 2,002).

**TB Localized body part**	**Treatment outcome of the patient**		**Chi square**
	**Successful treatment**	**Un-successful treatment**	
Lymphadenopathy	272 (85.5)	46 (14.5)	0.10
Pleural Effusion	133 (85.3)	23 (14.7)	
Lung	1,246 (89.6)	145 (10.4)	
Uncategorized EPTB	123 (89.8)	14 (10.2)	
Total	1,774 (88.6)	228 (11.4)	

### Trends of the defaulter, died and transferred out pediatric tuberculosis

**Figure 1:**
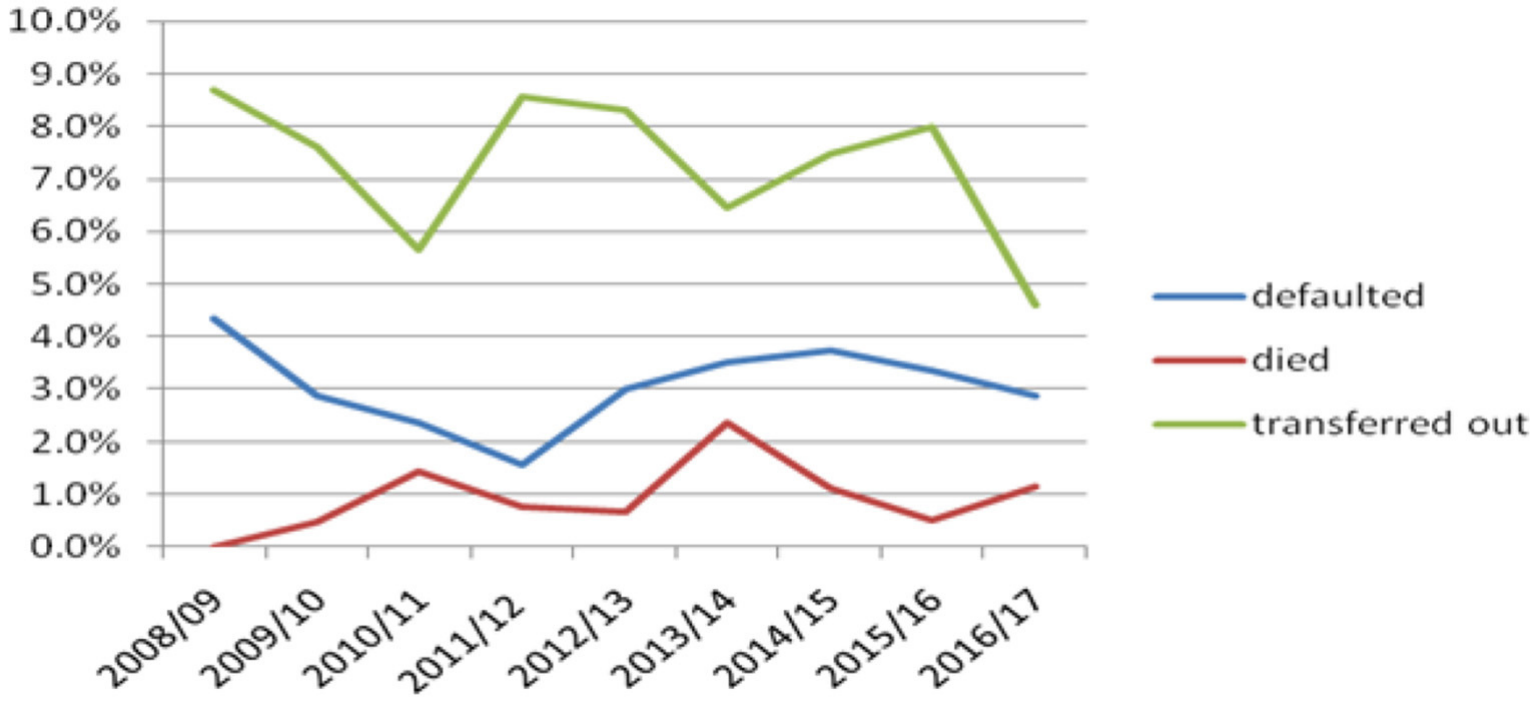
Trends of defaulter, died and transferred out children with TB attending treatment in selected health institutions of eastern Ethiopia, 2017.

There were a higher number of children transferred out of the health institutions from 2008/09 to 2016/17, with variable trends yearly. The number of defaulted children declined from 2008/09 to 2011/12, while it started to increase from 2011/12 to 2014/15. The trend of death was also variable, with a higher number of children dying in 2010/11 and 2013/14. There was an increase in the trend from 2011/12 to 2013/14 (Figure 1).

### Factors associated with treatment outcome

In bivariable analysis variables including sex, age, residence, types of TB, and HIV sero status were significant at a *p*-value <0.025 and considered as candidates for multivariable analysis. In multivariable analysis age and HIV sero status remained statistically significant at a *p*-value <0.05.

Being males in sex were 29% less likely to have a successful treatment outcome than females [AOR: 0.71, 95% CI = (0.53, 0.96)]. And those with HIV-positive sero status were 49% less likely to have a successful treatment outcome than those with unknown HIV sero status [AOR: 0.51; 95% CI = (0.29, 0.90)] (Table 5).

**Table 5:** Factors associated with treatment outcome of tuberculosis among children with TB attending Treatment in selected health institutions in eastern Ethiopia, 2017 (*n* = 2,002).

**Variables**		**Treatment outcome**		**Crude odds ratio (95 % Confidence interval)**	**Adjusted odds ratio (95 % Confidence interval)**
		**Successful = 1774 (88.6%) *N* (%)**	**Un-Successful =228 (11.4%) *N* (%)**		
Sex	Male	1,074 (87.5)	154 (12.5)	0.74 (0.55,0.99)	0.71 (0.53,0.96)*
	Female	700 (90.4)	74 (9.6)	1	1
Age (in years)	0–4	450 (87.4)	65 (12.6)	0.72 (0.4,1.2)	0.74 (0.44,1.24)
	5–9	616 (88.5)	80 (11.5)	0.8 (0.49,1.31	0.86 (0.52,1.42)
	10–14	496 (89)	61 (11)	0.84 (0.51,1.41)	0.85 (0.51,1.42)
	15–17	212 (90.6)	22 (9.4)	1	1
Residence	Urban	1,723 (88.5)	224 (11.5)	0.60 (0.22,1.69)	0.63 (0.23,1.77)
	Rural	51 (92.7)	4 (7.3)	1	1
Types of TB	SPTB	153 (92.2)	13 (7.8)	1.84 (0.99,3.39)	1.72 (0.93,3.19)
	SNPTB	1,091 (89.1)	133 (10.9)	1.27 (0.95,1.71)	1.26 (0.94,1.70)
	EPTB	529 (86.6)	82 (13.4)	1	1
Category of TB	New	1,769 (88.6)	227 (11.4)	ND	
	Re treatment	5 (83.3)	1 (16.7)		
HIV sero status	Positive	84 (82.4)	18 (17.6)	0.54 (0.31,0.92)	0.51 (0.29,0.90)*
	Negative	809 (88.1)	109 (11.9)	0.85 (0.64,1.13)	0.86 (0.64,1.14)
	Unknown	881 (89.7)	101 (10.3)	1	1

^*^p <0.05; ND, not done.

## Discussion

Children are susceptible to infection with *Mycobacterium tuberculosis* in the community and are at greater risk of progressing to active disease when infected at a very young age (2). A total of 2002 TB patients were included in this study with smear-positive pulmonary tuberculosis (SPPTB) (8.3%), smear-negative pulmonary tuberculosis (SNPTB) (61.1%), and extrapulmonary tuberculosis (EPTB) (30.5%). The findings of SPPTB were similar to the study conducted in Addis Ababa, Ethiopia (9.6%) (9), but higher than reports from Arsi, Ethiopia (12.3%) (8), and Iran (11). The SNPTB findings were higher than reports from Ethiopia; Arsi (54.0%) (8), and Addis Ababa (43.0%) (9).

The overall EPTB findings were comparable to Arsi, Ethiopia (33.1%) (8), and Rio de Janeiro Brazil (28.1%) (12). But lower than compared to the report from Addis Ababa, Ethiopia (47.4%) (9). And higher compared to previous studies conducted in the United State of America (USA) (22%) (13), and Pakistan (24.8%) (14). There was a high magnitude of lymph node TB (52.1%) among extrapulmonary TB. This was comparable to the finding reported from Greece (47%) (15). But higher than reports from Colombia (40.6%)(16), Tehran Iran (7%) (17), Italy, Rome (15.4%) (18) and Rio de Janeiro Brazil (40.3 %) (12). In the current study, Pleural effusion was the second most common EPTB (25.5%). This result was in line with the study findings reported from Athens, Greece (26.5%) (15) and Rio de Janeiro Brazil (27%)(12). The above differences might be due to the different diagnostic skills of health professionals, laboratory diagnostic tools, and the child's clinical presentation. The most significant challenges in most countries' TB control programs are difficulties in TB diagnosis in children (19). The report also indicates a proportion of TB cases were diagnosed due to non-response to antibiotics, which can delay the diagnosis of tuberculosis in children (20).

In this study, central nervous system (CNS) or Miliary TB cases were not reported. This result was different from the previous studies finding reported from Netherlands about 2.9% CNS or Miliary TB cases (21) and 11% CNS TB cases from Rome, Italy (18). This difference might be in the current study about one fifth of the children with EPTB were uncategorized into different groups, and sever form of TB like CNS or Miliary TB might be fall under the category of uncategorized EPTB. The other possible reason could be due to the difference of laboratory diagnosis and study duration.

In the current study, there was a higher magnitude of PTB (69.4%). This was similar to studies conducted in Ethiopia; Addis Ababa (52.6%) (9) and Arsi (54%) (8). But this was different from a Zaire study (59.0%) (22). Most of the PTB patients in this study have SNPTB findings. This was similar to Addis Ababa, Ethiopia study (9). Due to atypical presentation, many cases of pediatric TB may be misdiagnosed (23). Most children with TB also present with primary rather than secondary (cavitary lesions). This might have a low acid-fast bacilli load. In addition, because young children do not produce sputum for smear microscopy, they are diagnosed based on clinical and chest x-ray evidence.

In this study, there was a higher number of males (61.3%) diagnosed and treated with TB. This was similar to a study conducted in India (24). But, this was different from studies conducted in Addis Ababa, Ethiopia (9), Iran (19) and Israel (25). Concerning age, a higher number of cases (64.4%) were reported in children under the age of 10 in this study. This was similar to a study conducted in Israel (25). However, this study finding was different from that reported in Iran with higher pediatric TB above 10 years old (19). It was also reported that half percentage of under 10 years of age with TB were reported from the study conducted in Addis Ababa, Ethiopia (9). Of the under 10 children, the age groups 5–9 were highly affected in this study. This was similar to the study in Iran (19). This was different from the report with a higher magnitude of under 5 TB from a Zaire study (22). There are reports that indicate young children are at a higher risk of developing active tuberculosis.

In this study, the overall treatment success rate was 88.6%. This study's findings may be overestimated because the treatment outcomes of those who defaulted and transferred out were excluded. However, this finding was slightly higher than reports from Addis Ababa, Ethiopia (85.5%) (9), and Logos, Nigeria (79.2%) (26). This was lower than reports from Iran (91.7%) (19), Israel (97.8%) (25), and Russia (95.1%)(27). These differences may be due to differences in the study area, the magnitude of TB and HIV, parental/guardian awareness of TB, socio-cultural, economic, availability and accessibility of health facilities, management of TB or treatment monitoring criteria, and other factors.

In the current study, the rate of mortality among children with TB was 0.9%. This was similar to the report from Russia (0.9%) (27). But lower than reports from Ethiopia; Addis Ababa (3.3%) (9), Southern (5.8%) (28) and Arsi (5.3%) (8), and from other Africa studies conducted in Botswana (10.5%) (29), Malawi (17%) (30) Kinshasa, Zaire (1.4%) (22), and Asia; Iran (2.2%) (19). However, the default rate was 2.9%. This was higher than the report from Russia (0.5%) (27) and slightly lower than Addis Ababa, Ethiopia (3.8%) (9) and Kinshasa, Zaire (7.0%) (22). The treatment failure rate was 0.1%. This was lower than in Kinshasa, Zaire (2.5%) (22), and Russia (1.5%) (27). The highest death and default rates were reported among males, ages 0–4 and 15–17 years with EPTB and with unknown HIV status. This was different from a report of significantly higher mortality only among under-five children and those with HIV co-infection conducted in Addis Ababa, Ethiopia (9). Besides, males were less likely to have a successful treatment outcome than females. However, this was different from the study conducted in Iran (19).

In this study, those with HIV positive sero status were less likely to have a successful treatment outcome. This was similar to studies conducted in Addis Ababa, Ethiopia (9), Cote d'Ivore (31) Malawi (30), and the metropolitan area of Rio de Janeiro, Brazil (12). Those HIV positive individuals might have low immunity status, which can be co-infected with a number of pathogens with varying clinical manifestations. This might be responsible for their poor treatment adherence and/or outcome.

The treatment outcome between smear positive and smear negative was similar in this study. However, those smear positive children were more likely to have a poor treatment outcome in Addis Ababa, Ethiopia (9). Other studies have found that having smear positive PTB is associated with a favorable response to treatment (32); however, it is possible to conclude that children with SPPTB are at risk of failure or death due to the disease's advanced cavity formation.

This study also assessed the body part affected by TB with treatment outcome. It found that there was no difference in treatment outcome of TB among different subgroups of EPTB (TB lymphadenitis and pleural effusion). This was in line with the study conducted in Nigeria (33). But this different with study conducted in Kinshasa Zaire which reported low treatment rate completion among children with TB lymph node (67.8%) (34). The difference might due to difference in professional assessment capacity and tool used for the assessment of PTB and EPTB. From previous study conducted in Netherlands' found that those patients with CNS and Miliary TB had high unfavorable outcome (21). This might be because of their immature immune systems children appear to be more vulnerable to disseminated tuberculosis and tuberculosis meningitis with higher mortality and poor treatment outcomes (19, 35).

In this study, the magnitude of treatment success was higher in the older age group, relatively lower in under five age group. This was similar to the studies conducted in Addis Ababa, Ethiopia (9) and Iran (19). In addition, this was supported by the previous study conducted in Netherlands found that those patients with children aged <5 years had unfavorable outcome (21). Because of their immature immune systems, under five children appear to be more vulnerable to disseminated tuberculosis and tuberculosis meningitis, with higher mortality and poor treatment outcomes (19, 35). Despite the fact that CNS TB was not detected in this study, other studies have briefly discussed CNS TB management. The results of studies conducted in Netherland (21) and Denmark (36) indicated that CNS and miliary TB should be a concern in the management of TB as they had the highest mortality risk of any TB form. According to a study conducted in India, CNS TB had the most negative treatment outcomes and the highest death rate (37). In England and Wales, the proportion of children who completed treatment was lower among children with severe disease (miliary tuberculosis (68 %), and meningitis (60 %) (38). According to the study was conducted in Europe children with tuberculous meningitis, of 104 children with complete outcome data, 9.6% (10/104) died, and 47.1% (49/104) recovered fully (39). In Brazil, the percentage of children lost to follow-up was 9% and seven children (1%) died because of TB (including the child with MDR-TB). Among these children one child had TB meningitis (12).

## Limitation of the study

This study cannot get information on the treatment outcome of those transferred out. This might under estimate the treatment outcome of the children. In addition, this study lacks the classification of CNS and miliary TB among the group of EPTB because of almost one five of EPTB were not categorized in to different groups of TB. Parents/guardians' knowledge on the importance of TB treatment for their child, other children's factors like nutritional status and treatment compliance or adherence were not assessed.

## Conclusion

The treatment success rate was higher than the recent WHO report. Those with HIV seropositive status were less likely to have a successful treatment outcome. Therefore, efforts should be made by each health institution in eastern Ethiopia by giving emphasis to male and HIV positive individuals. This study will also recommend further study which will identify the different types TB based on the organ of localization with their treatment outcome.

## Data availability statement

The original contributions presented in the study are included in the article/supplementary material, further inquiries can be directed to the corresponding author.

## Ethics statement

The studies involving human participants were reviewed and approved by Institutional Health Research Ethics Review Committee of Haramaya University, College of Health and Medical Sciences (IHRERC/078/2017). Permission letter was obtained from each health institution managers.

## Author contributions

FW and ZT conceived and designed the study. FW, ZT, HM, TT, AAR, FT, AA, MN, BJG, FM, IMA, HB, and ET had participated in collecting scientific literature, critical appraisal of articles for inclusion, analysis, and interpretation of the findings. FW drafted the manuscript and prepared the manuscript for publication. All authors have read and approved the final version of the manuscript.

## Funding

Fund for data collection for this research was covered by Haramaya University Research Affair.

## Conflict of interest

The authors declare that the research was conducted in the absence of any commercial or financial relationships that could be construed as a potential conflict of interest.

## Publisher's note

All claims expressed in this article are solely those of the authors and do not necessarily represent those of their affiliated organizations, or those of the publisher, the editors and the reviewers. Any product that may be evaluated in this article, or claim that may be made by its manufacturer, is not guaranteed or endorsed by the publisher.
